# Differential effects of pesticides on dioxin receptor signaling and p53 activation

**DOI:** 10.1038/s41598-023-48555-x

**Published:** 2023-12-01

**Authors:** Myriam Fauteux, Nadia Côté, Sandra Bergeron, Alexandre Maréchal, Luc Gaudreau

**Affiliations:** Département de Biologie, Université de Sherbrooke, Sherbrooke, QC Canada

**Keywords:** Molecular biology, Risk factors

## Abstract

As modern agricultural practices increase their use of chemical pesticides, it is inevitable that we will find a number of these xenobiotics within drinking water supplies and disseminated throughout the food chain. A major problem that arises from this pollution is that the effects of most of these pesticides on cellular mechanisms in general, and how they interact with each other and affect human cells are still poorly understood. In this study we make use of cultured human cancer cells to measure by qRT-PCR how pesticides affect gene expression of stress pathways. Immunoblotting studies were performed to monitor protein expression levels and activation of signaling pathways. We make use of immunofluorescence and microscopy to visualize and quantify DNA damage events in those cells. In the current study, we evaluate the potential of a subset of widely used pesticides to activate the dioxin receptor pathway and affect its crosstalk with estrogen receptor signaling. We quantify the impact of these chemicals on the p53-dependent cellular stress response. We find that, not only can the different pesticides activate the dioxin receptor pathway, most of them have better than additive effects on this pathway when combined at low doses. We also show that different pesticides have the ability to trigger crosstalk events that may generate genotoxic estrogen metabolites. Finally, we show that some, but not all of the tested pesticides can induce a p53-dependent stress response. Taken together our results provide evidence that several xenobiotics found within the environment have the potential to interact together to elicit significant effects on cell systems. Our data warrants caution when the toxicity of substances that are assessed simply for individual chemicals, since important biological effects could be observed only in the presence of other compounds, and that even at very low concentrations.

## Introduction

Pesticides are a group of chemicals that are widely used in agriculture to prevent the infestation of cultured fields with unwanted weeds, insects, or fungi^1^. These chemicals are often used in combination, resulting in a cocktail of pesticides being released in the environment. In fact, there are several ways through which pesticides can spread from their initial site of intended usage: wind drift^2^, runoff of water contaminated by pesticides^3^, volatilization^4^, drainage^5^, and infiltration^6^. Consequently, humans become inadvertently exposed to pesticides through food and water consumption. Moreover, when pesticides are evaluated for their toxicity, they are mostly evaluated as single agents. However, in the environment, they are almost always found in combination. Their effect(s) while in combination are thus not well known.

Mammals are affected at the cellular and organismal levels by the presence of xenobiotics in their environment. These responses include changes in DNA methylation patterns and telomere length as well as changes in hormonal levels^7–10^. At the cellular level, one of the first molecular consequences of xenobiotics is the activation of the dioxin receptor, also known as the aryl hydrocarbon receptor (AhR)^11, 12^. Since many xenobiotics can produce DNA lesions, it is conceivable that a second pathway, would be triggered when cells are exposed to certain of these chemicals^13^.

Many environmental pollutants, such as polycyclic aromatic hydrocarbons (PAHs) and pesticides are metabolized by cytochrome P450 enzymes such as CYP1A1 and CYP1B1^11, 14^. The major transcription factor involved in the regulation of *CYP1* gene expression upon exposure to xenobiotics is AhR^15^. As a matter of fact, it has been shown in AhR^-/-^ mice that CYP1A1 was not induced when treated with TCDD (2,3,7,8-tetrachlorodibenzo-*p*-dioxin) while it was activated in AhR^+/+^ and AhR^+/−^ mice^16^. AhR is a ligand-activated receptor of the basic helix-loop-helix/Per-Arnt-Sim (bHLH/PAS) family^17, 18^. Typically, AhR is sequestered in the cytoplasm and upon ligand binding,^14^, is translocated into the nucleus where it heterodimerizes with its partner ARNT. The complex then recognizes xenobiotic response elements (XREs) within the regulatory regions of target genes in order to activate transcription^19, 20^.

A number of active ingredients within pesticide formulations act as ligands for AhR^21, 22^. For instance, a previous report has focussed on the characterization of AhR agonistic activity of more than 200 pesticides using a cell line that stably expresses an AhR responsive luciferase reporter gene. They showed that 11 pesticides (chlorpyrifos, diuron, prochloraz, acifluorfen-methyl, bifenox, isoxanthion, quinalphos, chlorpropham, diethofencarb, propanil, and linuron) behaved as AhR agonists. Similarly, a screen for putative activators of AhR using a chemical-activated luciferase gene expression (CALUX) assay confirmed that chlorpyrifos but also prochloraz and iprodione are bona fide AhR agonists since they were all capable of activating *CYP1A1* transcription^22^.

Some xenobiotics, for example benzo(a)pyrene, are metabolized by CYP1A1 and CYP1B1, two cytochrome P450 enzymes, as well as an epoxide hydrolase that converts the compound into an even more toxic molecule, benzo(a)pyrene-7,8-dihydrodiol-9,10-epoxide^23^. This compound can cause adducts in DNA by binding the exocyclic N2-position of guanine. If these DNA adducts remain unrepaired, they can block cell division and cause mutations^24^.

Another way through which CYP1 enzymes induced by the AhR pathway may cause DNA damage is through the enzymatic modification of estrogens. Indeed, CYP1A1 and CYP1B1^25–27^ can metabolize 17β-estradiol (E2) into 2-hydroxyestradiol (2-OHE_2_), and 4-hydroxyestradiol (4-OHE_2_), respectively^28^. Previous reports have shown that 2-OHE_2_ can inhibit cell cycle progression by activating amongst others, p53 or the CHK1 checkpoint kinase whereas 4-OHE_2_ possesses genotoxic properties^29–34^. Interestingly, studies have shown that the estrogen-regulated transcription factor ERα is involved in a crosstalk with the AhR xenobiotic-response pathway at the level of transcriptional regulation of cytochrome p450 genes. ERα is a member of nuclear receptors which are ligand-activated transcription factors^35^. It regulates the expression of genes in response to estrogen^36, 37^. Thus, ERα selectively represses *CYP1A1* but not the *CYP1B1* gene in cells treated with TCDD, a dioxin that is a well-established ligand for AhR^35, 38–41^. Our laboratory has further shown that Dnmt3B, a DNA methyltransferase, is involved in the specific repression of *CYP1A1*. In fact, Dnmt3B specifically methylates the XRE3 response element upstream of the *CYP1A1* gene, which causes its transcriptional repression^42^. As a result, the cellular CYP1B1/CYP1A1 ratio is modified, and consequently the ratio of the estrogen metabolites is also modified leading to an accumulation of genotoxic 4-OHE_2_.

Thus, when exposed to xenobiotics such as dioxins, and potentially a large number of pesticides, can activate the AhR pathway, which promotes detoxification but it could also paradoxically create DNA damage, either directly or indirectly. Accordingly, another potential cellular response to xenobiotics exposure could be the DNA damage response. When cells are insulted by genotoxic agents, they will activate pathways that block cell cycle progression and repair DNA damage^43^. If the damage is too extensive, cells will undergo programmed cell death^44^. One of the cellular responses to DNA damage is the activation of a checkpoint that is controlled by the p53 tumor suppressor^45–47^. This pathway can regulate the expression of genes involved in major cellular pathways such as cell cycle control, apoptosis and DNA repair in order to maintain proper genome integrity^46, 48, 49^. For instance, to investigate how the cell cycle is affected by DNA damage, one can investigate levels of certain marker proteins such as ATM (p-S1981), p53(p-S15), and p21 that are all involved in the p53 pathway, as well as γ-H2A.X, which marks DNA damage foci (38). ATM (p-S1981) is an autophosphorylation mark that is rapidly triggered by DNA damage. The ATM kinase is also responsible, to a certain extent, of the phosphorylation of serine 15 on p53^50–52^, which is a phosphorylation mark typical of p53 activation^53, 54^. p21 is a major cyclin-dependent kinase inhibitor implicated in cell cycle arrest^13, 47^.

In this work we wanted to evaluate the propensity of different pesticides to interact positively or negatively with each other to activate the AhR and p53 signaling pathways. To achieve this, five active ingredients found within commercially sold pesticides were chosen based on two criteria: (1) their chemical structure, and (2) their use in agricultural practices. For the structures, we chose pesticides having at least one aromatic cycle, which may bind to the AhR ligand-binding domain^55, 56^. We also chose pesticides that are currently banned in the OECD (Organization for Economic Co-operation and Development) countries but still widely used in Canada and the US. The first one, bromoxynil, is an herbicide that inhibits photosynthesis and affects oxidative phosphorylation. It is typically used on wheat, barley and oat cultures^57^. LD50 for bromoxynil in rats is 190 mg/kg for an oral absorption^58^. The second and the third one, carbaryl and chlorpyrifos, are both insecticides that inhibit acetylcholinesterase. They are both widely used on some cultures such as corn and apples^57^. LD50 for chlorpyrifos in rats is 135 mg/kg for an oral absorption and LD50 for carbaryl in rats is 300 mg/kg for an oral absorption^58^. The fourth one, linuron, is an herbicide that inhibits photosynthesis. It is commonly used on potatoes, oats, carrots, and soya^57^. LD50 for linuron in rats is 4000 mg/kg for an oral absorption^58^. The last one, thiabendazole, is a fungicide that inhibit fungal microtubular function^59^. It is usually used on citrus fruits and corn seeds to prevent putrefaction^57^. LD50 for thiabendazole in rats is over 3330 mg/kg for an oral absorption^58^. Pesticides are found within the environment at different concentrations which can vary greatly depending on the place and country in which they are found (soil, underground water, surface water). For instance, in Canada, drinking water was monitored for the presence of carbaryl and the data reported a maximum concentration of 0.005 ppb (parts per billion) (24.6 pM), while in the USA the maximum concentration was 0.16 ppb (0.79 nM)^74^. For chlorpyrifos, in 1998, the EPA reported concentrations of 0.1 µg/L to 0.4 µg/L (0.2 nM to 1.14 nM) in surface water^78^. Between 2003 and 2004, Harman-Fetcho and al. reported concentrations ranging from 0.30 to 1.89 ng/L, mean 1.5 ng/L (4,27 pM) in surface water^79^. For linuron, the EPA (environmental protection agency) estimates that 6.9 to 60 µg/L (27 nM to 0.2uM) of linuron are found in the environment. The maximum concentration of linuron reported in surface waters in California with agricultural watersheds is 0.71 µg/L (2.9 nM)^75^. In Costa Rico, thiabendazole is used at concentrations ranging from 1–100 µg/L (4.97 nM to 0.497 µM) have been found in surface waters. According to the FQPA Index Reservoir Screening Tool (FIRST), the concentrations following exposure to thiabendazole vary between 3.80 ppb (18 nM) for surface water and 0.62 ppb (3 nM) for groundwater^76^. According to Health Canada (2020), the maximum concentration of bromoxynil residues allowed on food is 0.9 ppm which is equivalent to 3.25 µM. Health Canada establishes the maximum acceptable concentration (MAC) in drinking water at 30 µg/L, therefore 0.1 µM^77^.

Pesticides are commonly used in combination, however there are very few studies that have addressed the effects of these combinations on cells. Using these pesticides in combination, we were able to show that all of the pesticides could activate the AhR pathway, while only a few could activate p53. Most of the pesticides, in combination, exhibit a strong effect on the induction of the *CYP1A1* gene, while no significant effect could be observed on the DNA damage response when combined together at low concentrations. The significance of these findings is discussed.

## Materiel and methods

### Chemicals and reagents

17β-Estradiol (E2), daunorubicin, camptothecin, Tween-20, paraformaldehyde and all active ingredients of pesticides, bromoxynil, carbaryl, chlorpyrifos, linuron and thiabendazole, were purchased from Sigma-Aldrich (Sigma-Aldrich, Saint-Louis, MO, USA). Bovine serum albumin (BSA) and sucrose were purchased from Bioshop (Bioshop, Burlington, ON, CA).

### Cell culture and treatments

MCF-7 and HCT116 cells from ATCC were maintained in DMEM medium (Wisent, St-Jean-Baptiste, QC, CA) supplemented with 10% FBS (Wisent) and 1% Pen/Strep (Invitrogen, Thermo fisher scientific, Waltham, MA, USA) at 37 °C in a humid environment containing 5% CO_2_. For chemical treatment of cells, cells were transferred into six-well plates with 500,000 cells per well with 2 mL of DMEM medium. After 24 h of culture, medium was replaced with DMEM medium containing active ingredients of pesticides diluted in DMSO. After 24 h of treatment, cells were lysed and lysates kept at −20 °C until further processing.

Cross-talk experiments were performed as described previously^42^ between AhR and ERα, after the first 24 h of culture, culture media was replaced with phenol red-free DMEM (Wisent) containing 10% FBS that was previously stripped using activated charcoal (Sigma-Aldrich) according to the manufacturer’s instructions and 1% Pen/Strep (Invitrogen). After 48 h in stripped DMEM (Wisent), culture media was replaced with stripped DMEM containing active ingredients of pesticides diluted in DMSO 100% and supplemented or not with 10 nM 17β-estradiol diluted in DMSO 100%. After 24 h of treatment, cells were lysed and kept at − 20 °C until further processing.

### qRT-PCR

Human *CYP1A1*, *CYP1B1* and *p21* mRNAs were quantified by qRT-PCR using *36B4* (Acidic ribosomal phosphoprotein P0) as an internal control. Total RNA was extracted from cultured cells using EZ-10 DNAaway RNA Miniprep Kit (BioBasic, Cerdarlane, Burlington, ON, CA). The reverse transcription was made in Whatman Biometra Tgradient. 300 ng of RNA was reversed transcribed with M-MuLV reverse transcriptase (Enzymatics, Qiagen, Hilden, Germany). RT-qPCR runs were performed in CFX Connect Real-Time PCR Detection System (Bio-Rad, St-Laurent, Qc, CA). Two µL of cDNA was added to each qPCR reaction mix (8 µL), containing 0.4 µL of 10 µM for each primer, 5 µL of advanced qPCR mastermix with supergreen LO-ROX (Wisent) and 2.2 µL of water. The following protocol was used: an initial step at 95 °C for 2 min, followed by 40 cycles at 95 °C for 5 s, 60 °C for 30 s with a final standard dissociation protocol to obtain the melting profiles. Data were acquired using the CFX Manager software. Primers are listed in Table 1.

**Table 1:** qRT-PCR primers.

Fwd 36B4	CGACCTGGAAGTCCAACTAC
Rev 36B4	ATCTGCTGCATCTGCTTG
Fwd CYP1A1	TGAACCCCAGGGTACAGAGA
Rev CYP1A1	GGCCTCCATATAGGGCAGAT
Fwd CYP1B1	AACGTACCGGCCACTATCAC
Rev CYP1B1	CCACGACCTGATCCAATTCT
Fwd p21	GGAGACTCTCAGGGTCGAAA
Rev p21	GGATTAGGGCTTCCTCTTGG

### Immunoblotting

MCF-7 cells were treated or not with chemicals, washed with cold PBS, harvested and resuspended in RIPA buffer (50 mM Tris–HCl, pH 7.5, 150 mM NaCl, 1% Triton X-100, 0.5% Na-deoxycholate, 0.2% SDS, 1 mM DTT, 1X PMSF) and disrupted by passing five times through a 23G1 needle. Lysis was performed for 1 h at 4 °C with continuous agitation. The cell solution was then sonicated under 50% power for 5 s. The lysate was cleared by centrifugation at 14 000 rpm for 10 min at 4 °C. All the antibodies used are listed in Table 2.

**Table 2:** Antibodies.

	Manufacturers	Catalog numbers	Applications	Primary antibody dilution	Secondary dilution
Actin	Sigma	A2066	WB	1:1000	1/20,000 rabbit
ATM	Bethyl	A300-299A	WB	1:1000	1/20,000 rabbit
ATM-pS1981	Abcam	ab81292	WB	1:1000	1/20,000 rabbit
H2A.X-pS139	Cell signaling	9718	IF	1:1000	1/20,000 rabbit
p53	Santa Cruz	sc-6243	WB	1:1000	1/20,000 rabbit
p53-pS15	Cell signaling	9284S	WB	1:1000	1/20,000 rabbit

### Immunofluorescence

MCF-7 cells were grown on coverslips. Cells were fixed with 3% paraformaldehyde in 2% sucrose solution for 15 min at room temperature. Then, cells were permeabilized with ice-cold 0.5% Triton-X100 in PBS on ice for 5 min, blocked in 3% BSA and 0.05% Tween-20 in PBS prior to incubation with primary antibodies. After 4 washes with 0.05% Tween-20 in PBS, cells were incubated with secondary antibodies. DAPI solution (1 μg/mL) was used for nuclei staining. Coverslips were mounted onto microscope slides using ProLong^®^ Diamond Antifade Mountant (Life Technologies, Thermo fisher scientific, Waltham, MA, USA). All the antibodies used are listed in Table 2.

Images were taken using a Zeiss AxioObserver Z1 with a 63X/1.4 oil objective, a Zeiss Axiocam 506 camera and the Zeiss Zen 2 software. Image pre-processing and segmentation were performed with the open source software CellProfiler (version 2.2)^60^.

### Cell proliferation assays

MCF-7 NucLight (gift of Pr. Viktor Steimle) cells in DMEM media supplemented with FBS 10% were seeded at 1 × 10^4^/well in a 96 well plate for 24 h and treated with the active ingredients. The experiment was performed in triplicate. Cells were placed in the IncuCyte S3 live-cell analysis instrument (Sartorius, Oakville, ON, CA) with a 10× objective in a standard cell culture incubator at 37 °C. Four images per well were collected every 12 h in both phase contrast and fluorescence for 6 days. Green count was analyzed using IncuCyte software.

### Statistical analyses

The data are presented as mean ± standard deviation (SD) and were analyzed with GraphPad Prism software. Comparisons between two variants was analyzed by t-test. Comparisons between several groups were performed using one-way variance analysis (ANOVA); Dunnett’s multiple comparison method was employed. Comparisons between two independent variant was analyzed with two-way ANOVA; Šídák's multiple comparisons test was performed. The value p < 0.05 was considered statistically significant.

## Results

### Induction of CYP1A1 by active ingredients found in pesticides

**Figure 1:**
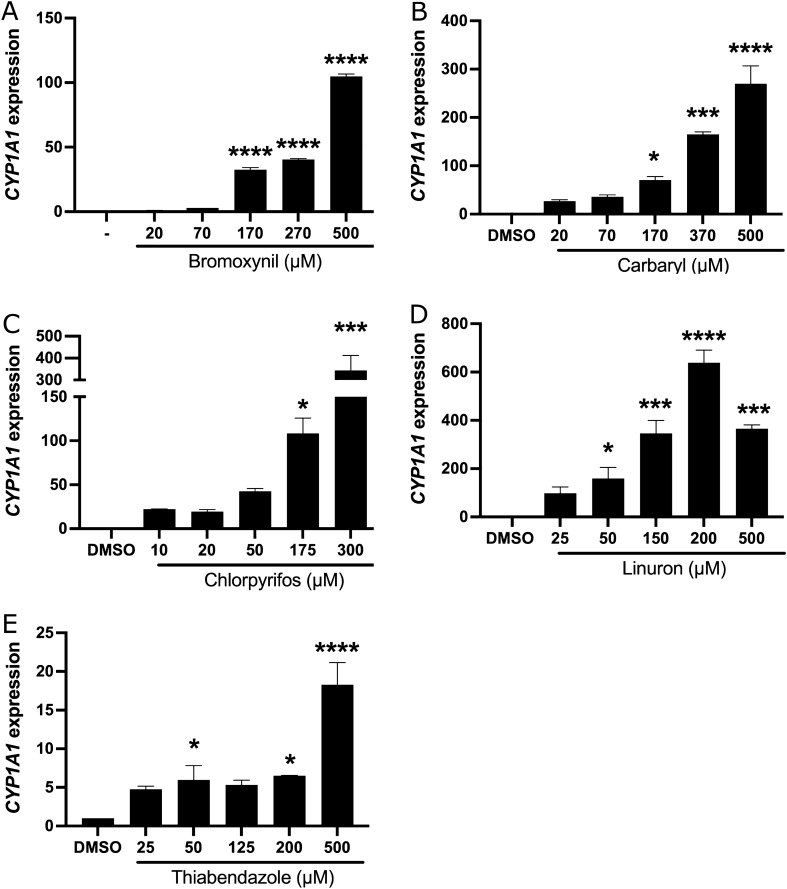
Pesticides activate *CYP1A1* expression in MCF-7 cells. *CYP1A1* mRNA levels were quantified by qRT-PCR in MCF-7 cells grown in DMEM media supplemented with FBS 10% for 24 h, and then treated with (**A**) bromoxynil, (**B**) carbaryl, (**C**) chlorpyrifos, (**D**) linuron and (**E**) thiabendazole for 24 h while "–" corresponds to DMSO treated samples. Single biological replicates are shown here because the total activation fold of *CYP1A1* was not constant in all experiments. However, strong activation of *CYP1A1* transcription was observed in all experiments by all pesticides tested. Experiments were performed three times independently and the mean expression of *CYP1A1* is shown in Supplementary Table 1. Values are presented as mean ± S.D *p < 0.05, **p < 0.01, ***p < 0.001, ****p < 0.0001 by one-way ANOVA with Dunnett’s multiple comparisons test compared to DMSO.

Since some active ingredients within pesticides were previously shown to act as AhR ligands^21, 22^, we first decided to investigate their ability to activate *CYP1A1* transcription through the AhR pathway in the MCF-7 breast cancer cell line. This cell line was chosen because it has functional AhR, ERα, and p53 signaling pathways, and several groups have used them in similar studies^35–37, 42^. To assess this, we measured the expression of the *CYP1A1* gene (a commonly used marker to measure the extent of induction of the AhR pathway^11, 61, 62^) by qRT-PCR after treatment of cells with various concentrations of all the active ingredients for 24 h. While testing different time points, we observed that 24 h was optimal for the majority of our tested pesticides. The results (Fig. 1) show that bromoxynil, carbaryl, chlorpyrifos, linuron, and thiabendazole can all significantly activate *CYP1A1* to various extents as their respective concentrations are increased. In order to verify that what we observe in MCF-7 cells is not unique to this cell type we performed similar experiments in HCT116 colon cancer cells. Although the activation potential of the different pesticides is not as robust as in MCF-7 cells, we can still observe significant activation levels of *CYP1A1* with the different pesticides (Supplementary Fig. 1). Single biological replicates are shown here and two additional experiments are shown in Supplementary Fig. 2. The mean expression of *CYP1A1* of all three experiments are shown in Supplementary Table 1. In the main figures we show our expression data using the *36B4* internal qPCR control but we also used GeNorm software to choose other internal controls, namely *TFIIB* and *ACTB*. In all cases tested we obtained identical results as with *34B4* so we pursued all other experiments using the latter (Supplementary Fig. 3).

### A combination of active ingredients can activate CYP1A1 even at low concentrations

As mentioned previously, pesticides are commonly used in combination in agricultural fields thus leading to a variety of different chemicals in the environment. Some active ingredients have been significantly studied as single agents^21, 63–65^, but the effects of a combination of active ingredients are still largely unknown. This led us to investigate how a combination of active ingredients can affect the AhR signaling pathway. Throughout our experiments where we use combinations of pesticides, we have tried to use concentrations of pesticides that alone do not significantly activate *CYP1A1* to establish a “measurable” range of potential additive or better than additive effect on transcriptional output.

**Figure 2:**
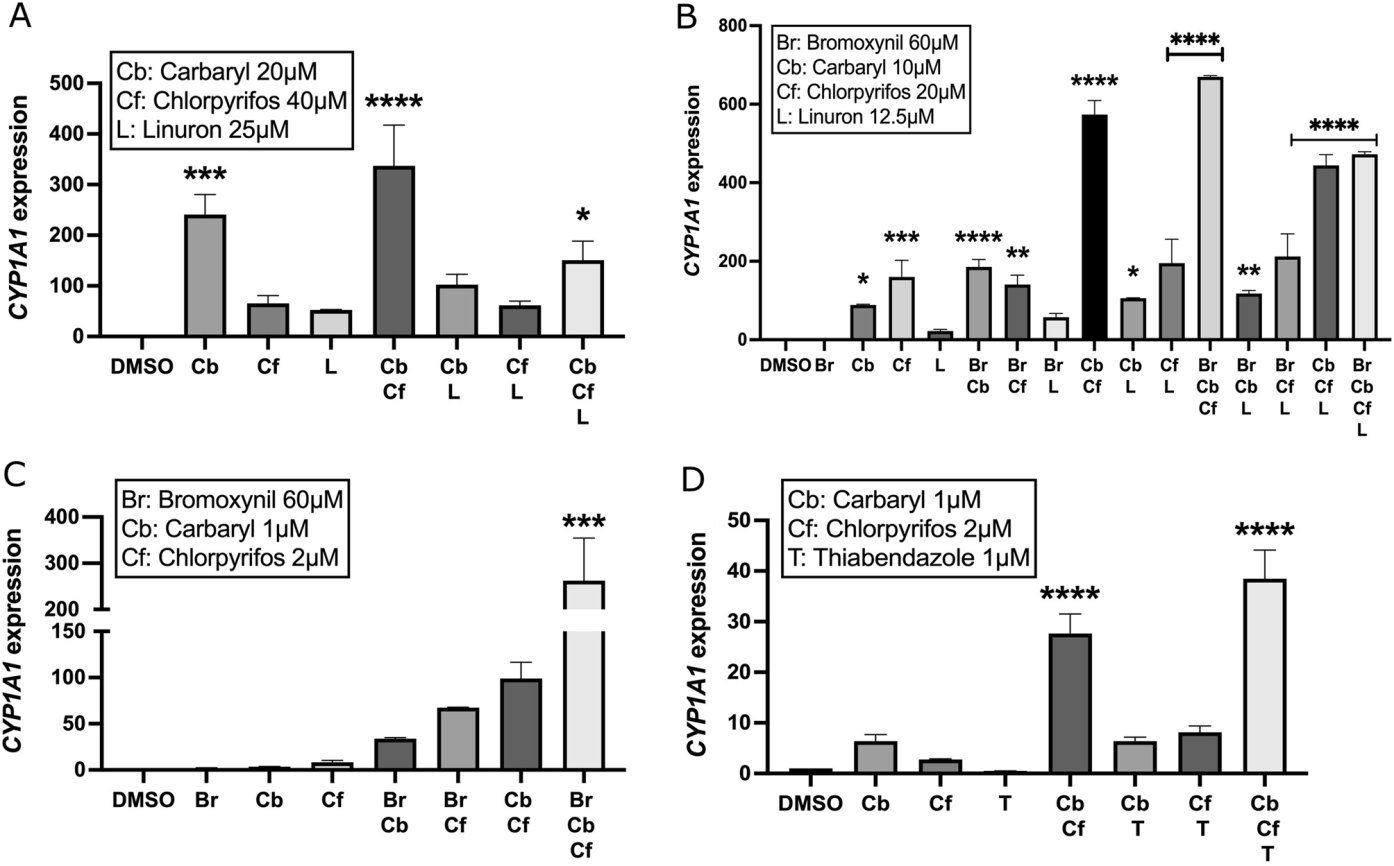
Combinations of pesticides can have a synergistic effect on the expression of *CYP1A1. CYP1A1* mRNA levels were quantified by qRT-PCR in MCF-7 cells grown in DMEM media supplemented with FBS 10% for 24 h and then treated with every possible combination containing (**A**) 20 μM carbaryl, 40 μM chlorpyrifos and 25 μM linuron, (**B**) 60 μM bromoxynil, 10 μM carbaryl, 20 μM chlorpyrifos and 12.5 μM linuron, (**C**) 1 μM carbaryl, 2 μM chlorpyrifos and 60 μM bromoxynil and (**D**) 1 μM carbaryl, 2 μM chlorpyrifos and 1 μM thiabendazole for 24 h while "–" correspond to DMSO treated samples. Single biological replicates are shown here because the total activation fold of *CYP1A1* was not constant in all experiments. However, strong activation of *CYP1A1* transcription was observed in all experiments by all pesticides tested. Experiments were performed three times independently and the mean expression of *CYP1A1* in shown in Supplementary Table 2. Values are presented as mean ± S.D *p < 0.05, **p < 0.01, ***p < 0.001, ****p < 0.0001 by one-way ANOVA with Dunnett’s multiple comparisons test compared to DMSO.

First, a mix of three active ingredients containing 20 µM carbaryl, 40 µM chlorpyrifos, 25 µM linuron, and all possible combinations of these active ingredients was tested (Fig. 2A). As previously shown in Fig. 1, all active ingredients alone activate *CYP1A1* at a higher dose, between 10× and 25× higher than the one used for this combination. In all combinations tested that contained linuron, the induction of *CYP1A1* was lower than the addition of the effect of each pesticide alone. This led us to believe that linuron has a negative effect on the induction potential of the other pesticides tested. In fact, only the combination without linuron has an additive effect on the activation of the AhR pathway.

Since addition of linuron to the other chemicals appeared to cancel out their induction potential on *CYP1A1*, we next aimed to create another experimental setup by adding 60 µM bromoxynil while having the concentration of the other components leading to a final mix containing 60 µM bromoxynil, 10 µM carbaryl, 20 µM chlorpyrifos, and 12.5 µM linuron. The idea here was to verify if adding bromoxynil could alleviate the inhibitory effect of linuron in that setting. 60 mM was chosen since that concentration is able to synergize with 12.5 μM linuron (Fig. 2B). Once again, all possible combinations and every active ingredient alone were tested on MCF-7 cells (Fig. 2B). As expected, all active ingredients alone led to the induction of *CYP1A1.* Our results further demonstrate that some combinations lead to an additive effect whereas other combinations lead to better than additive effects. In fact, the combination of chlorpyrifos and linuron, chlorpyrifos and bromoxynil, linuron and carbaryl, chlorpyrifos, linuron and bromoxynil and finally linuron, carbaryl and bromoxynil all led to an additive activation of the AhR pathway. Moreover, the combination of chlorpyrifos and carbaryl, linuron and bromoxynil, carbaryl and bromoxynil and finally chlorpyrifos, carbaryl and bromoxynil all led to significant and better than additive activation of the AhR pathway. However, addition of linuron to these combinations still had an inhibitory effect on *CYP1A1* expression levels, and those two combinations are chlorpyrifos, carbaryl and linuron as well as carbaryl, chlorpyrifos, linuron and bromoxynil. All things considered, we can conclude that linuron still has an inhibitory effect on the other chemicals’ ability to activate *CYP1A1*, but this effect is less pronounced when the concentration of linuron is lower. In addition, we were able to demonstrate that no matter what the combination of active ingredients were, the effect on the activation of the AhR pathway is always greater than the effect of a single active ingredient.

Next, we wanted to test what happens when we exclude linuron of the mixture because of its potential inhibitory effect on the other chemicals while lowering the concentrations by tenfold of the carbaryl and chlorpyrifos chemicals. To achieve this, MCF-7 cells were treated for 24 h with all possible combinations of 60 µM bromoxynil, 1 µM carbaryl and 2 µM chlorpyrifos and every active ingredient alone (Fig. 2C). While *CYP1A1* induction was virtually undetectable when exposed to single agents, it was strongly enhanced by combinations of two pesticides and this was even more pronounced when cells were treated with a cocktail of the three compounds at low concentrations. Our results show that every combination, without exception, all led to better than additive activation of the AhR pathway. Interestingly, combinations that were previously shown to activate AhR in better than additive manner are even pronounced when their concentrations are reduced. Likewise, the combination of chlorpyrifos and bromoxynil that was previously shown to activate AhR in an additive manner activates AhR in a strong synergistic manner under these conditions.

Finally, since the concentration of bromoxynil is higher than the concentration used for the other pesticides, we wanted to test what happens when we substitute bromoxynil with thiabendazole. As with our other experiments, MCF-7 cells were treated for 24 h with all possible combinations of 1 µM thiabendazole, 1 µM carbaryl and 2 µM chlorpyrifos and every active ingredient alone (Fig. 2D). Again, all combinations led to better than additive activation of the AhR pathway except for the combination of chlorpyrifos and thiabendazole which led to an additive activation of *CYP1A1,* and carbaryl combined to thiabendazole that did not show any significant effect.

Taken together, our results show that several pesticides have the ability not only to act as AhR agonists, but also when at low concentration, can interact together and strongly induce AhR signaling.

### ERα represses CYP1A1 in the presence of estradiol in cells treated with active ingredients either alone or in combination

**Figure 3:**
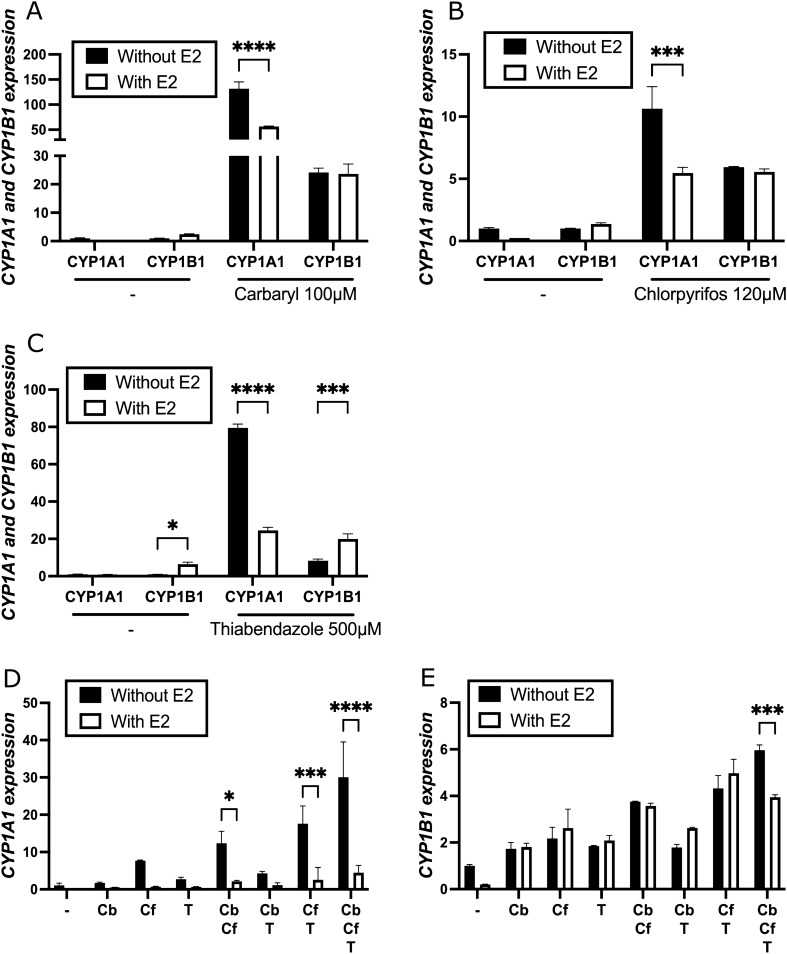
ERα specifically represses *CYP1A1* expression in MCF-7 cells treated with pesticides. *CYP1A1* and *CYP1B1* mRNA levels were quantified by qRT-PCR in MCF-7 cells grown in estrogen-free media supplemented with FBS 10% for two days and then treated with (**A**) 100 mM carbaryl, (**B**) 120 mM chlorpyrifos, (**C**) 500 mM thiabendazole, and every possible combination of 1 μM carbaryl, 2 μM chlorpyrifos and 1 μM thiabendazole (**D**) *CYP1A1* and (**E**) *CYP1B1* with and without estradiol for 24 h while "–" correspond to DMSO treated samples. Single biological replicates are shown here because the total activation fold of *CYP1A1* was not constant in all experiments. However, strong activation of *CYP1A1* transcription was observed in all experiments by all pesticides tested. Experiments were performed three times independently and the mean expression is shown in Supplementary Table 3. Values are presented as mean ± SD *p < 0.05, **p < 0.01, ***p < 0.001, ****p < 0.0001 by one-way ANOVA with Dunnett’s multiple comparisons test compared to DMSO.

It was previously shown that the ERα transcription factor selectively represses *CYP1A1,* but not *CYP1B1* in the presence of estradiol in MCF-7 cells treated with TCDD, a dioxin that is an AhR ligand^41, 42^. That suggests that there is a modification in the *CYP1A1/CYP1B1* ratio and therefore that there is a change in the estrogen metabolite ratio leading to an accumulation of genotoxic 4-OHE2. We aimed to investigate if the pesticides mentioned above would have the same effect as TCDD^29, 41^ on the ability to elicit ERα-dependent repression of *CYP1A1*. To test this, we measured the expression of *CYP1A1* and *CYP1B1* by qRT-PCR after treatment of cells with thiabendazole, carbaryl and chlorpyrifos, and a combination of those three pesticides either in presence or absence of estradiol for 24 h in MCF-7 cells pre-grown in hormone-free media for 48 h (Fig. 3). Linuron was not tested because of its inhibitory effect, and bromoxynil also was not tested because of its low activation potential at 20 μM, the concentration used in pesticide combination experiments.

In the presence of estradiol, *CYP1A1* was repressed in all treatments compared to the cells grown in the absence of estradiol. On the other hand, the levels of *CYP1B1* did not change significantly either in the presence or in the absence of estradiol except for the treatment with thiabendazole and the combination of thiabendazole and carbaryl, where *CYP1B1* was slightly increased. That suggests that thiabendazole affects a different pathway than carbaryl and chlorpyrifos. One explanation could be that thiabendazole, in the presence of E2, could activate the expression of an activator of *CYP1B1* that has no effect on *CYP1A1*. It also suggests that thiabendazole induces a bigger change in the ratio of *CYP1A1*/*CYP1B1*, therefore potentially leading to a higher accumulation of 4-OHE2.

Taken together, our results suggest that the tested active ingredients allow ERα to selectively repress *CYP1A1*, as it does when cells are exposed to the dioxin TCDD^40–42^, and they also suggest that a combination of pesticides can mediate similar effects.

### Specific pesticides can activate the p53-dependant cellular stress pathway

**Figure 4:**
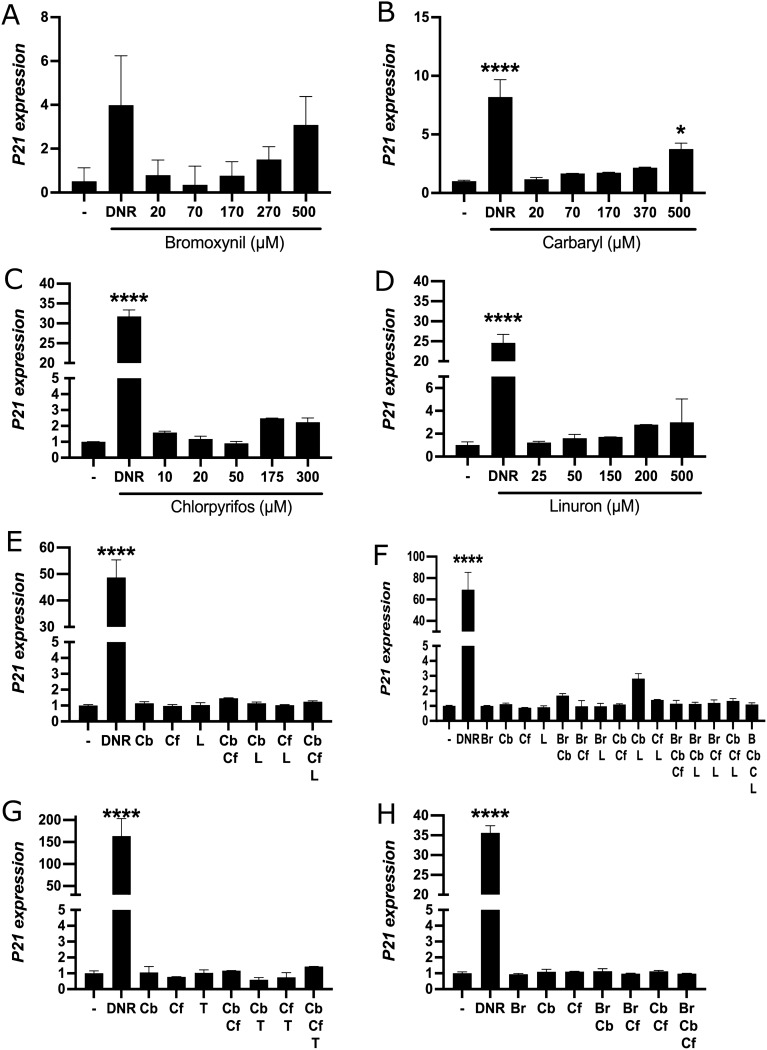
Carbaryl, linuron and bromoxynil can activate the p53-dependent cellular stress pathway. MCF-7 cells were grown in DMEM media supplemented with FBS 10% for 24 h and then treated with daunorubicin 250 nM (DNR), camptothecin 1 μM (CPT), chlorpyrifos 300 μM (Cf), carbaryl 500 μM (Cb), linuron 500 μM (L), bromoxynil 500 μM (B), thiabendazole 500 μM (T) and four combinations containing 20 μM carbaryl, 40 μM chlorpyrifos and 25 μM linuron (Cf,Cb,L), or 60 μM bromoxynil, 10 μM carbaryl, 20 μM chlorpyrifos and 12.5 μM linuron (Cf,Cb,L,B), or 1 μM carbaryl, 2 μM chlorpyrifos and 60 μM bromoxynil (Cf, Cb, B) and 1 μM carbaryl, 2 μM chlorpyrifos and 1 μM thiabendazole (Cf, Cb,T) for 24 h. Cells were subsequently lysed and protein extracts were analyzed by immunoblotting against the indicated proteins while "–" correspond to DMSO treated samples. Values are presented as mean ± SD *p < 0.05, **p < 0.01, ***p < 0.001, ****p < 0.0001 by one-way ANOVA with Dunnett’s multiple comparisons test compared to DMSO.

**Figure 5:**
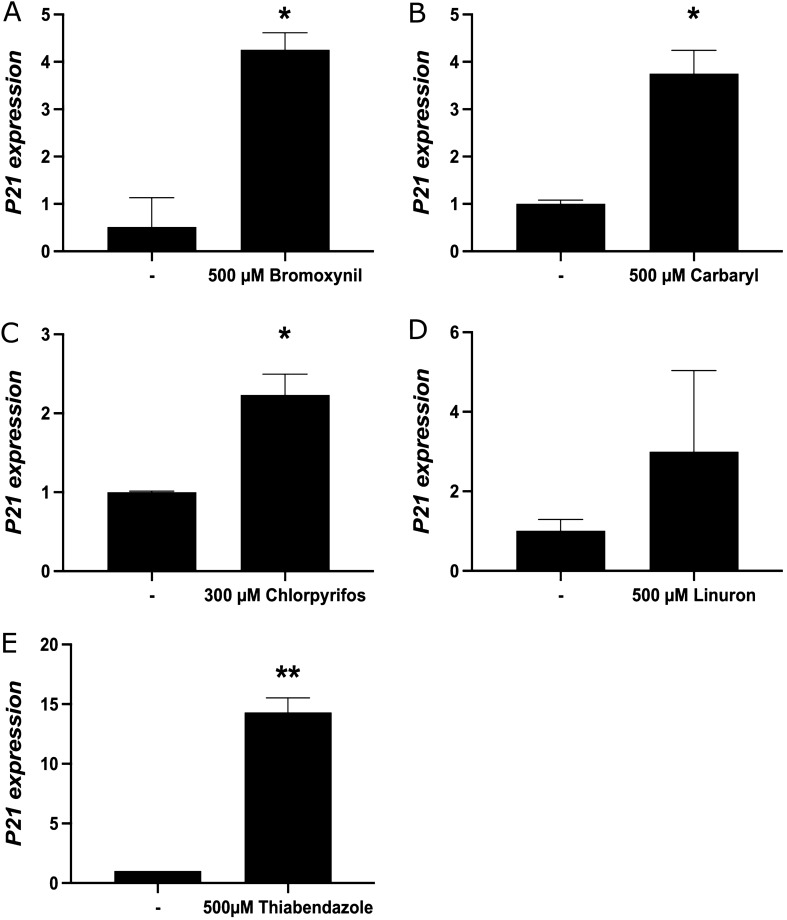
Pesticides can activate *p21* expression in MCF-7 cells. *p21* mRNA levels were quantified by qRT-PCR in MCF-7 cells grown in DMEM media supplemented with FBS 10% for 24 h and then treated with (**A**) bromoxynil (500 μM), (**B**) carbaryl (500 μM), (**C**) chlorpyrifos (300 μM), (**D**) linuron (500 μM), and (**E**) thiabendazole (500 μM) for 24 h while "–" correspond to DMSO treated samples. Single biological replicates are shown here because the total activation fold of *CYP1A1* was not constant in all experiments. However, strong activation of *CYP1A1* transcription was observed in all experiments by all pesticides tested. Experiments were performed three times independently. Brightness/cropping of the images was performed in order to improve clarity of the image. Supplementary Table 4 shows the mean expression of *p21* in MCF-7 cells treated with every single pesticides, and Suppelmenrary Table 5 shows the mean expression of *p21* in MCF-7 cells treated with every combinations of pesticides. Values are presented as mean ± SD *p < 0.05, **p < 0.01, ***p < 0.001, ****p < 0.0001 by one-way ANOVA with Dunnett’s multiple comparisons test compared to DMSO.

Certain xenobiotics have previously been documented to induce a p53-dependent cellular stress response^13^. We wanted to test whether our selected pesticides could also individually induce a p53-dependent stress response but also more importantly, we wanted to test if the pesticides, when at low concentrations and in a mixture (conditions that strongly activate the AhR pathway), would also induce that stress response. To address this, we first monitored levels of serine 15 (S15) phosphorylation within p53, which is an event that stabilizes p53 and also serves as a marker for its activation^66^. Levels of ATM serine 1981 (S1981) phosphorylation were also monitored, which is the kinase that phosphorylates p53 at serine 15 in response to DNA damage^50, 51^. Thus, we carried out immunoblotting experiments using protein extracts from MCF-7 cells treated with individual pesticides as indicated at concentrations that efficiently induce *CYP1A1* alone (Fig. 4, left panel), or in combination but at much lower concentration where we observe better than additive effects on *CYP1A1* activation (right panel). We also used the DNA damaging agents daunorubicin and camptothecin as positive controls in our experiments since they can both efficiently induce the p53 pathway^54, 67–69^. Our results show that carbaryl, linuron, and bromoxynil can significantly trigger p53 S15 and ATM S1981 phosphorylation, whereas chlorpyrifos and thiabendazole cannot. In contrast, p53 and ATM total protein levels were unchanged by our treatments. None of the mixed pesticides could induce p53 S15 or ATM S1981 phosphorylation. We next decided to further substantiate our results obtained with p53 S15 and ATM S1981 phosphorylation, and measured expression of the p53 target gene *p21* under the same conditions as in Fig. 4 by qRT-PCR. We find that that all pesticides can activate *p21* to various extents with thiabendazole and bromoxynil being the best inducers (Fig. 5). It is thus conceivable in this case that both thiabendazole and chroryrifos can induce *p21* without triggering a DNA damage response. Finally, we measured the expression of *p21* in MCF-7 cells treated with every combinations of pesticides at low concentrations (Supplementary Table 5) and find no significant change in gene expression.

Taken together, our results suggest that it is possible certain pesticides can while others cannot strongly induce the AhR pathway without significantly inducing a robust p53/DNA damage cellular stress response when pesticides are at low concentrations.

### Carbaryl, linuron, and bromoxynil can cause DNA damage

**Figure 6:**
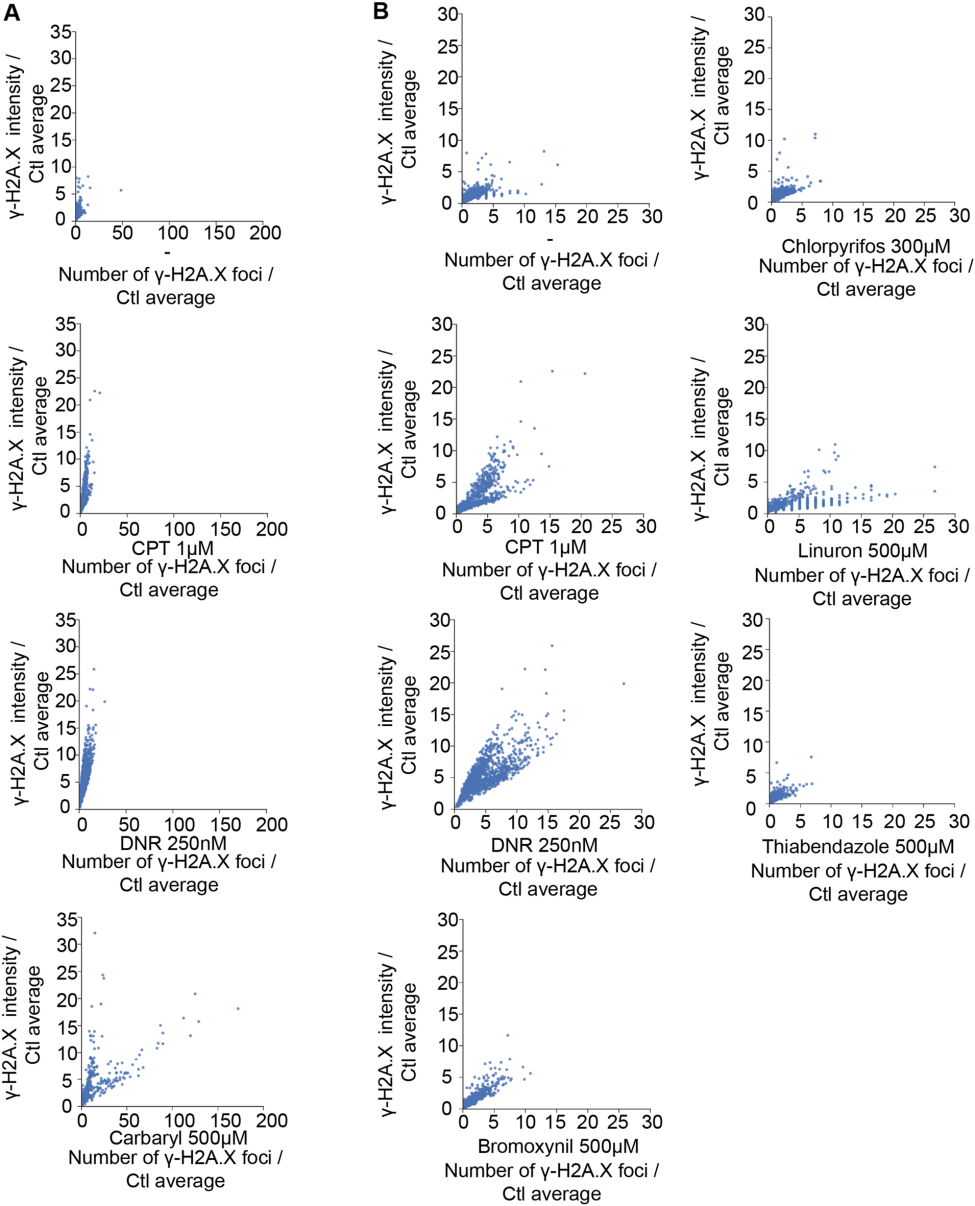
Carbaryl, linuron and bromoxynil cause DNA damage. γ-H2A.X foci were observed by immunofluorescence on MCF-7 cells grown in DMEM media supplemented with FBS 10% for 48 h and treated with (**A**) DMSO, camptothecin 1 μM (CPT), daunorubicin 250 nM (DNR) and carbaryl 500 μM, (**B**) DMSO, camptothecin 1 μM (CPT), daunorubicin 250 nM (DNR), bromoxynil 500 μM, chlorpyrifos 300 μM linuron 500 μM and thiabendazole 500 μM for 24 h while "-" correspond to DMSO treated samples. Results of DMSO, DNR and CPT are the same on both panels of this figure but with a different scale to facilitate comparison with bromoxynil, chlorpyrifos, linuron and thiabendazole.

Since certain active ingredients can induce the p53 cellular stress pathway, we wanted to verify whether they could also cause DNA damage, either alone or combined at low concentrations. To evaluate this, we checked for the presence of histone variant H2A.X phosphorylated on serine residue 139 (γ-H2A.X) foci in the nucleus, a well-established indicator of DNA damage, which is rapidly gaining traction as a biomarker in ecotoxicological assays of water supplies^70–72^. We carried out immunofluorescence experiments using MCF-7 cells treated with individual pesticides at concentrations that efficiently induce *CYP1A1* alone, or in combination but at much lower concentration where we previously observed synergistic *CYP1A1* activation (Fig. 6). The genotoxic agents daunorubicin and camptothecin were employed as positive controls in our experiment. Our results demonstrate that only carbaryl, linuron and bromoxynil (Fig. 6, Supplementary Fig. 4) cause significant DNA damage whereas chlorpyrifos, thiabendazole and all the mixes of pesticides at low concentrations cannot (Supplementary Fig. 5).

### Certain pesticides can affect cell proliferation

**Figure 7:**
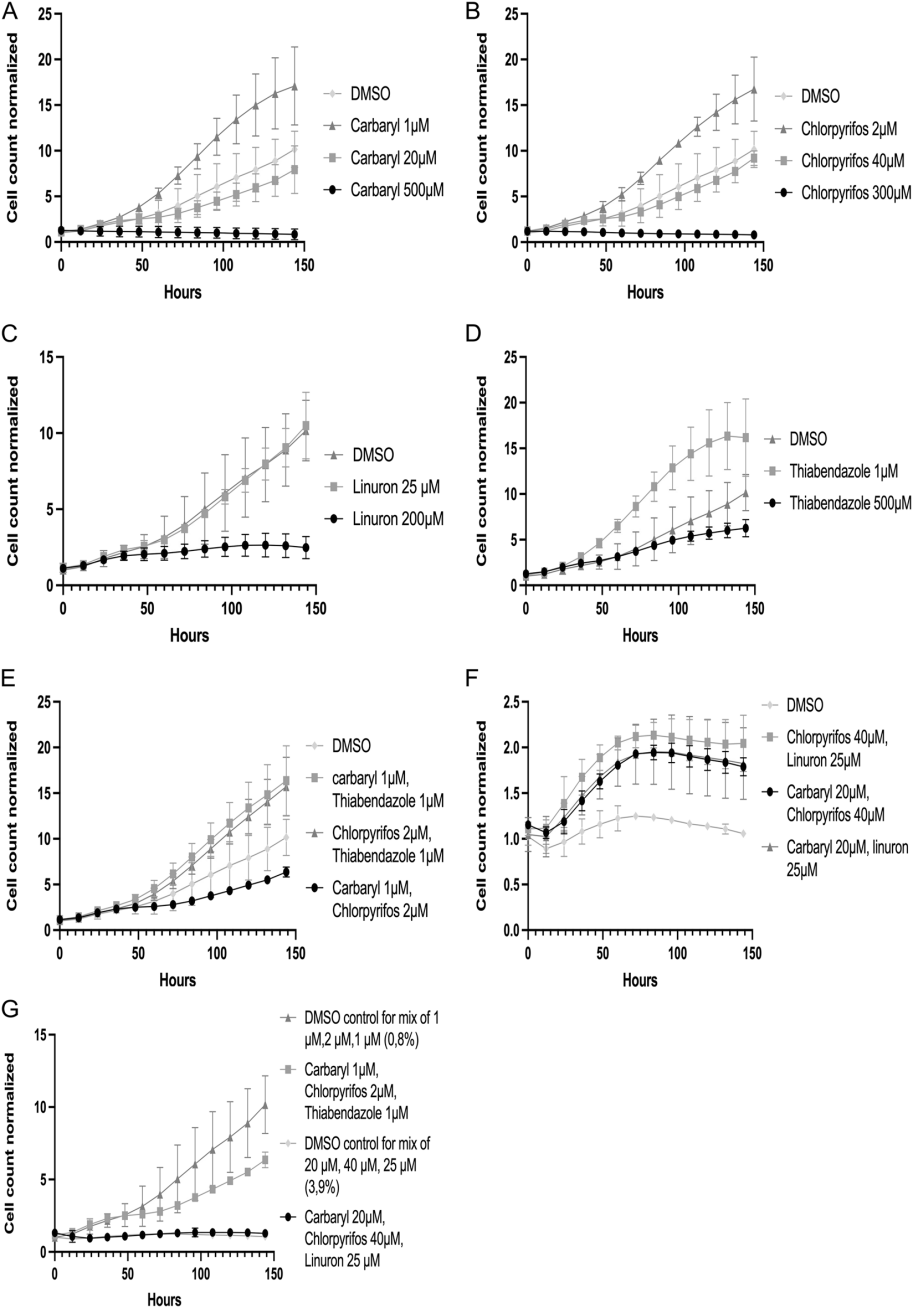
Certain pesticides can affect cell proliferation. Proliferation of MCF7 Nuclight cells normalized on DMSO (time 0 h) treated with different concentrations of (**A**) carbaryl, (**B**) chlorpyrifos, (**C**) linuron, (**D**) thiabendazole, (**E**) double combinations of 1 μM carbaryl, 2 μM chlorpyrifos, and 1 μM thiabendazole, (**F**) double combinations of 20 μM carbaryl, 40 μM chlorpyrifos, and 25 μM linuron, and (**G**) triple combinations of 1 μM carbaryl, 2 μM chlorpyrifos, 1 μM thiabendazole and 20 μM carbaryl, 40 μM chlorpyrifos, and 25 μM linuron for 6 days.

In order to again further gain insight into how our select pesticides affect cell mechanisms we monitored cell growth and proliferation upon treatment with pesticides for a period of over six days. Figure 7 shows that carbaryl, chlorpyrifos, linuron and thiabendazole all inhibit cell growth to various extents with carbaryl and linuron being the most effective (Fig. 7A–D). Figure 7E–G show that a combination of pesticides individually at a lower concentration can also inhibit cell growth. Taken together these results show that while all tested pesticides can clearly inhibit cell growth at a certain concentration, the cellular effects are likely not mediated by induction of the p53–p21 pathway because of the differential behaviour observed between the respective pesticides in this pathway and growth inhibition.

## Discussion

Our study reveals that five active ingredients found in commonly used pesticides in agricultural practices can efficiently induce *CYP1A1* gene expression. Since all of these chemicals thus constitute potential bona fide AhR ligands, we surmised, for example, that the activation potential of *CYP1A1* would be comparable whether we have 5× concentration of one ingredient (ligand), or 1× concentration of five different ingredients. In a more biologically relevant context, this prediction could then signify that tens, even hundreds of AhR ligands found at very low concentrations within the environment could have a very significant effect on AhR signaling when combined together. Accordingly, our results show that, when at low concentrations, several pesticides do not significantly activate AhR—as opposed to when they are at higher concentration—but when combined to other pesticides they can have a very significant effect on AhR activation. Depending on the concentration range that we used with our active ingredients, we either observed additive or synergistic effects on *CYP1A1* induction. However, in most experimental conditions tested, linuron tended to have an inhibitory effect on the potential of the other pesticides to exhibit better than additive effects. The mechanism by which this inhibition occurs remains to be determined, but it is conceivable that linuron somehow binds the ligand-binding domain of AhR more efficiently than the other pesticides, and exhibits a sort of ‘squelching’ effects. Squelching in this case would be defined as saturating the ligand binding domain of AhR with a very weak K_D_ such as no other pesticide can efficiently compete with that putative ligand, and thus subsequently prevent synergy by allowing multiple interactions between AhR and the transcriptional machinery. This however would need to be addressed in a more formal fashion but remains a worthy future research venue.

Another important issue that we wished to address pertained to whether or not different pesticides (or a mixture thereof) could also have the potential to perturb estrogen metabolism, as TCDD does^40–42^. Our findings reveal that indeed these different chemicals can inevitably lead to changes in the CYP1A1/CYP1B1 ratio. The impact of this scenario is significant since it suggests that a great deal of different pesticides within the environment have the potential to activate the AhR pathway, and even though certain combinations of these chemicals may not be directly genotoxic themselves, they may still increase the frequency of DNA damaging events by perturbing estrogen metabolism. This is in line with a study we have performed and shown that ERα can recruit the Dnmt3 DNA methyltransferase to direct repression of *CYP1A1* but not *CYP1B1*^42^. However, we observe that under conditions where our mixture of pesticides is diluted and acts synergistically to induce the AhR pathway, the p53-dependent cellular stress response is not significantly induced. In fact, even at higher concentrations, only carbaryl, linuron, and bromoxynil produce detectable DNA damage and induce p53 activation. It is also interesting to note that all pesticides have the ability to induce *p21* gene expression, even though chlorpyrifos and thiabendazole do not induce DNA damage *per s*e. That would suggest that some pesticides have the ability to induce *p21* within activating a DNA damage response, a scenario which could still result in the induction of cellular senescence, but that remains to be determined. Accordingly, all pesticides tested have the ability to inhibit cell growth at least to some extent at certain concentration, a result that emphasizes the fact that other cellular pathways could be affected by pesticide exposure. The concentration of pesticides that we used are 1000X greater than what can be detected in surface waters^73^. These results could mean that under conditions that most likely represent concentrations of xenobiotics found within the environment, we would not be able to detect a global and significant stress response within cells. We note however that most current measurement methods of cellular stress (*e.g.* activation of the p53 pathway) rely on massive global changes (such as protein phosphorylation) as measured by immunoblotting techniques etc. Those methods may not be well suited to detect less dramatic changes caused by lower concentrations of pesticides. It is thus likely that DNA damaging events do indeed occur under our experimental conditions but our analytical methods simply are not sensitive enough to detect the damage.

Taken together, we describe a system where different pesticides can have differential effects on cell signaling pathways. Moreover, these different chemicals have the ability to significantly affect cells when they are at low concentration. This paves the way for many future studies where we will focus on defining how xenobiotics contained either within water sources or agricultural soils, have the ability to perturb signaling pathways, such as the AhR pathway.

### Supplementary Information

Supplementary Information 1.

Supplementary Information 2.

Supplementary Information 3.

## Data Availability

The datasets used and/or analyzed during the current study are available from the corresponding author on reasonable request.

## Abbreviations

AhR: Aryl hydrocarbon receptor
ERα: Estrogen receptor alpha
E2: 17β-Estradiol
PAHs: Polycyclic aromatic hydrocarbons
HAHs: Halogenated aromatic hydrocarbons
XREs: Xenobiotic response elements
2-OHE_2_: 2-Hydroxyestradiol
4-OHE_2_: 4-Hydroxyestradiol
