# Supplementary figures and images for: Differential effects of pesticides on dioxin receptor signaling and p53 activation
Source: Sci Rep. 2023 Dec 1;13:21211. doi: 10.1038/s41598-023-48555-x (PMC10692357; doi:10.1038/s41598-023-48555-x)

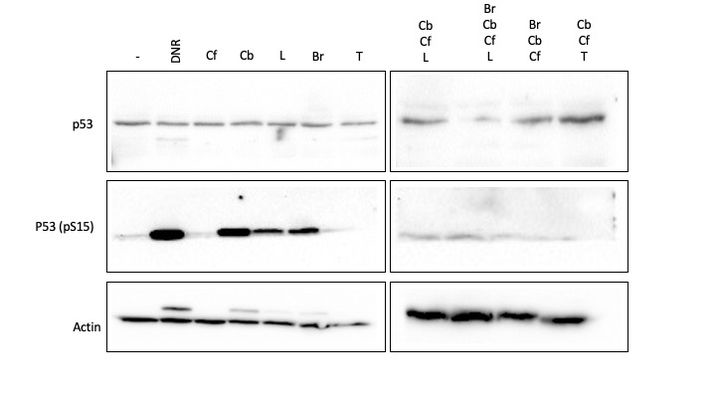

Supplement: Supplementary file 1 — Supplementary Information 1. [file 41598_2023_48555_MOESM1_ESM.tiff]

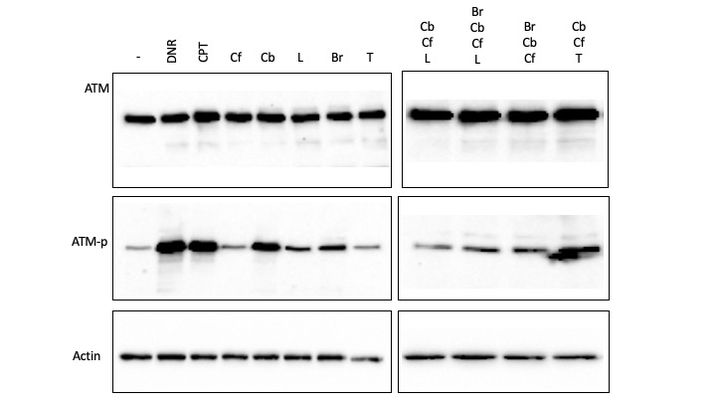

Supplement: Supplementary file 2 — Supplementary Information 2. [file 41598_2023_48555_MOESM2_ESM.tiff]
