# Supplementary material for: Differential effects of pesticides on dioxin receptor signaling and p53 activation
Source: Sci Rep. 2023 Dec 1;13:21211. doi: 10.1038/s41598-023-48555-x (PMC10692357; doi:10.1038/s41598-023-48555-x)
Supplement: Supplementary file 3 — Supplementary Information 3. [file 41598_2023_48555_MOESM3_ESM.docx]

**Differential effects of pesticides on dioxin receptor signaling and p53 activation**

Myriam Fauteux, Nadia Côté, Sandra Bergeron, Alexandre Maréchal, and Luc Gaudreau

**Additional files 1**

**Supplementary table 1. Mean expression of *CYP1A1* expression in MCF-7 cells treated with five different singles pesticides.**

| Treatment | Concentration | Mean *CYP1A1* expression |
| --- | --- | --- |
| Bromoxynil | Non-treated | 1 |
|  | 20 μM | 2.14 |
|  | 70 μM | 2.48 |
|  | 170 μM | 29.10 |
|  | 270 μM | 54.66 |
|  | 500 μM | 102.12 |
| Carbaryl | Non-treated | 1 |
|  | 20 μM | 29.35 |
|  | 70 μM | 51.75 |
|  | 170 μM | 77.61 |
|  | 270 μM | 137.14 |
|  | 500 μM | 192.94 |
| Chlorpyrifos | Non-treated | 1 |
|  | 20 μM | 11.31 |
|  | 70 μM | 19.40 |
|  | 170 μM | 66.48 |
|  | 270 μM | 159.88 |
|  | 500 μM | 439.33 |
| Linuron | Non-treated | 1 |
|  | 20 μM | 55.05 |
|  | 70 μM | 120.67 |
|  | 170 μM | 251.47 |
|  | 270 μM | 373.12 |
|  | 500 μM | 185.96 |
| Thiabendazole | Non-treated | 1 |
|  | 20 μM | 7.96 |
|  | 70 μM | 10.35 |
|  | 170 μM | 15.82 |
|  | 270 μM | 18.28 |
|  | 500 μM | 32.78 |

**Supplementary table 2. Mean expression of *CYP1A1* expression in MCF-7 cells treated with four different combinations of pesticides*.***

| Treatment | Combinaison | Mean *CYP1A1* expression |
| --- | --- | --- |
| Br (60 μM)  Cb (1 μM)  Cf (2 μM) | Non-treated | 1 |
|  | Br | 1.53 |
|  | Cb | 4.49 |
|  | Cf | 8.01 |
|  | Br Cb | 39.92 |
|  | Br Cf | 31.72 |
|  | Cb Cf | 25.29 |
|  | Br Cb Cf | 119.14 |
| Cb (20 μM)  Cf (40 μM)  L (25 μM) | Non-treated | 1 |
|  | Cb | 105.18 |
|  | Cf | 102.04 |
|  | L | 108.05 |
|  | Cb Cf | 396.72 |
|  | Cb L | 240.23 |
|  | Cf L | 166.60 |
|  | Cb Cf L | 223.54 |
| Br (60 μM)  Cb (10 μM)  Cf (20 μM)  L (12,5 μM) | Non-treated | 1.02 |
|  | Br | 54.06 |
|  | Cb | 50.21 |
|  | Cf | 119.04 |
|  | L | 10.07 |
|  | Br Cb | 166.24 |
|  | Br Cf | 162.40 |
|  | Br L | 54.25 |
|  | Cb Cf | 485.01 |
|  | Cb L | 153.62 |
|  | Cf L | 312.52 |
|  | Br Cb Cf | 657.77 |
|  | Br Cb L | 236.08 |
|  | Br Cf L | 338.83 |
|  | Cb Cf L | 364.02 |
|  | Br Cb Cf L | 583.77 |
| Cb (1 μM)  Cf (2 μM)  T (1 μM) | Non-treated | 1 |
|  | Cb | 12.84 |
|  | Cf | 11.01 |
|  | T | 2.92 |
|  | Cb Cf | 51.68 |
|  | Cb T | 29.98 |
|  | Cf T | 18.36 |
|  | Cb Cf T | 54.82 |

**Supplementary table 3. Mean expression of *CYP1A1/CYP1B1* in MCF-7 cells treated with single pesticides or in combination.**

| Treatment | Mean *CYP1A1* expression | Mean *CYP1B1* expression |
| --- | --- | --- |
| DMSO | 1 | 1 |
| DMSO + E2 | 0.29 | 2.76 |
| Cb 100 uM | 128.25 | 20.89 |
| Cb 100 uM + E2 | 67.09 | 23.34 |
| DMSO | 1 | 1 |
| DMSO + E2 | 0.24 | 1.13 |
| Cf 120 uM | 10.01 | 4.46 |
| Cf 120 uM + E2 | 5.33 | 4.79 |
| DMSO | 1 | 1 |
| DMSO + E2 | 1.33 | 5.44 |
| T 1500 uM | 45.10 | 5.57 |
| T 500 uM + E2 | 15.73 | 17.55 |
| DMSO | 1 | 1 |
| DMSO + E2 | 0.25 | 1.56 |
| Cb | 2.10 | 1.65 |
| Cb + E2 | 0.56 | 2.33 |
| Cf | 12.31 | 3.46 |
| Cf + E2 | 1.75 | 3.64 |
| T | 6.12 | 2.52 |
| T + E2 | 1 | 2.72 |
| Cb Cf | 22.16 | 5.31 |
| Cb Cf + E2 | 2.98 | 5.66 |
| Cb T | 9.67 | 3.54 |
| Cb T + E2 | 2.17 | 4.55 |
| Cf T | 27.21 | 5.78 |
| Cf T + E2 | 7.06 | 6.20 |
| Cb Cf T | 38.30 | 7.62 |
| CB Cf T + E2 | 8.96 | 8.12 |

**Supplementary table 4. Mean expression of *p21* in MCF-7 cells treated with every single pesticides.**

| Treatment | Concentration | Mean *p21* expression |
| --- | --- | --- |
| Bromoxynil | Non-treated | 1 |
|  | 20 μM | 1.20 |
|  | 70 μM | 0.98 |
|  | 170 μM | 2.40 |
|  | 270 μM | 4.57 |
|  | 500 μM | 6.76 |
| Carbaryl | Non-treated | 1 |
|  | 20 μM | 1.05 |
|  | 70 μM | 1.31 |
|  | 170 μM | 1.35 |
|  | 270 μM | 1.78 |
|  | 500 μM | 2.73 |
| Chlorpyrifos | Non-treated | 1 |
|  | 20 μM | 1.46 |
|  | 70 μM | 1.34 |
|  | 170 μM | 1.09 |
|  | 270 μM | 4.30 |
|  | 500 μM | 5.77 |
| Linuron | Non-treated | 1 |
|  | 20 μM | 1.20 |
|  | 70 μM | 1.52 |
|  | 170 μM | 2.13 |
|  | 270 μM | 2.80 |
|  | 500 μM | 3.62 |

**Supplementary table 5. Mean expression of *p21* in MCF-7 cells treated with every combinations of pesticides.**

| Treatment | Combinaison | Mean *p21* expression |
| --- | --- | --- |
| Br (60 μM)  Cb (1 μM)  Cf (2 μM) | - | 1 |
|  | Br | 0.84 |
|  | Cb | 1.14 |
|  | Cf | 0.93 |
|  | Br Cb | 0.95 |
|  | Br Cf | 0.94 |
|  | Cb Cf | 0.97 |
|  | Br Cb Cf | 0.92 |
| Cb (20 μM)  Cf (40 μM)  L (25 μM) | - | 1 |
|  | Cb | 0.80 |
|  | Cf | 0.85 |
|  | L | 0.84 |
|  | Cb Cf | 1.08 |
|  | Cb L | 1.03 |
|  | Cf L | 0.97 |
|  | Cb Cf L | 1.01 |
| Br (60 μM)  Cb (10 μM)  Cf (20 μM)  L (12,5 μM) | - | 1 |
|  | Br | 1.06 |
|  | Cb | 1.18 |
|  | Cf | 1.03 |
|  | L | 1.14 |
|  | Br Cb | 1.26 |
|  | Br Cf | 1.04 |
|  | Br L | 1.02 |
|  | Cb Cf | 1.32 |
|  | Cb L | 1.67 |
|  | Cf L | 1.35 |
|  | Br Cb Cf | 1.12 |
|  | Br Cb L | 1.14 |
|  | Br Cf L | 1.10 |
|  | Cb Cf L | 1.23 |
|  | Br Cb Cf L | 1.08 |
| Cb (1 μM)  Cf (2 μM)  T (1 μM) | - | 1 |
|  | Cb | 0.88 |
|  | Cf | 0.82 |
|  | T | 1.04 |
|  | Cb Cf | 0.60 |
|  | Cb T | 0.41 |
|  | Cf T | 0.68 |
|  | Cb Cf T | 0.77 |

**Supplementary figure 1. *CYP1A1* activation by pesticides in HCT116 colon cancer cells.**

*CYP1A1* mRNAs were quantified by qRT-PCR using *36B4* as an internal control. Total RNA was extracted from cultured cells using RNeasy Mini Kit (Qiagen) and reversed transcribed using M-MuLV reverse transcriptase (Enzymatics). Primers are listed in Table 1.

**Supplementary figure 2. Biological replicates of *CYP1A1* activation by pesticides in MCF7 cells.** *CYP1A1* mRNAs were quantified by qRT-PCR using *36B4* as an internal control. Total RNA was extracted from cultured cells using RNeasy Mini Kit (Qiagen) and reversed transcribed using M-MuLV reverse transcriptase (Enzymatics). Primers are listed in Table 1.

**Supplementary figure 3. *CYP1A1* activation by pesticides in MCF7 cells using *TFIIB* and *ACTB* as internal controls.** *CYP1A1* mRNAs were quantified by qRT-PCR using *TFIIB and ACTB* as internal controls. Total RNA was extracted from cultured cells using RNeasy Mini Kit (Qiagen) and reversed transcribed using M-MuLV reverse transcriptase (Enzymatics). Primers are listed in Table 1.

**
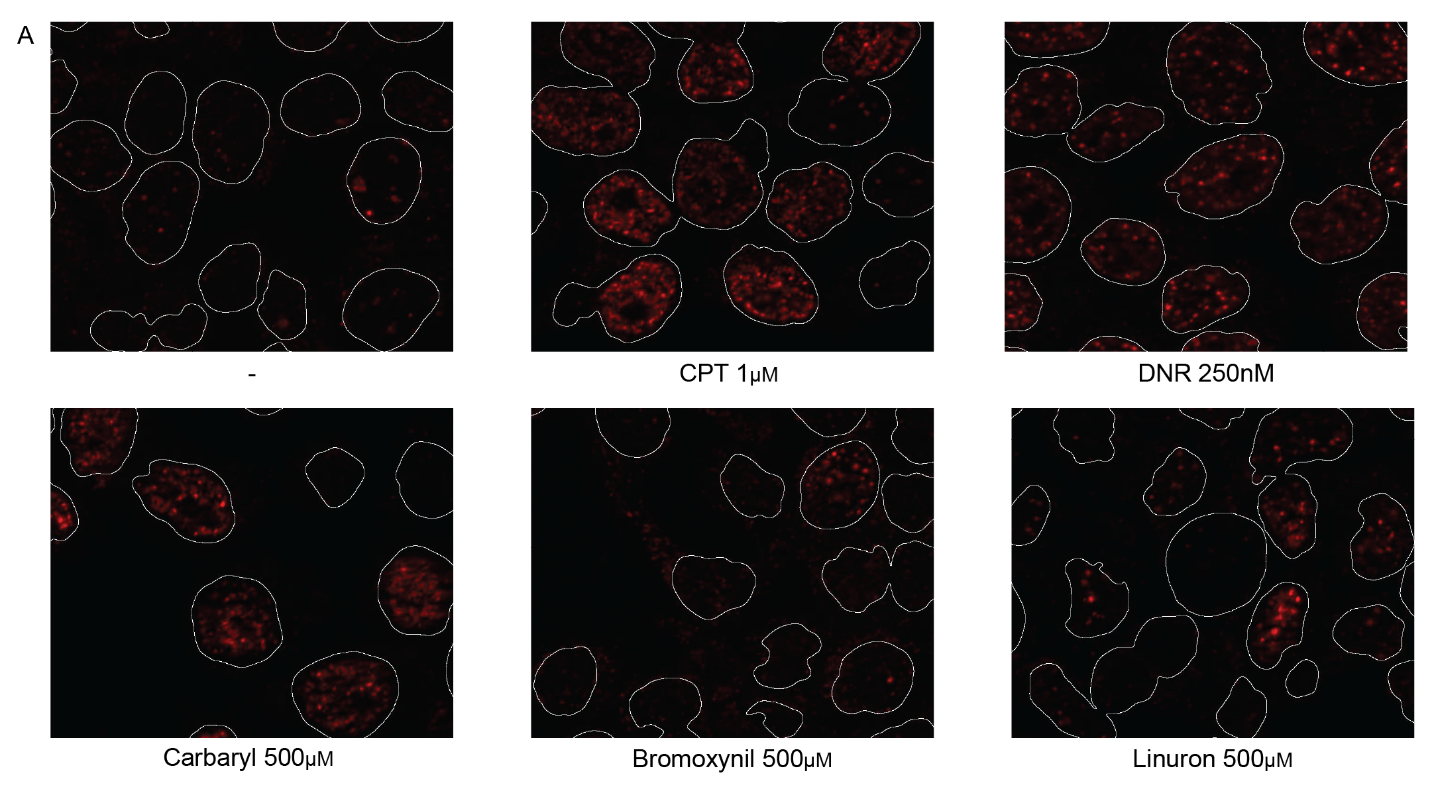
**

**Supplementary figure 4. Carbaryl, linuron and bromoxynil cause DNA damage.**

γ-H2A.X foci were observed by immunofluorescence on MCF-7 cells grown in DMEM media supplemented with FBS 10 % for 48h and treated with DMSO, camptothecin 1μM (CPT), daunorubicin 250nM (DNR), carbaryl 500μM, bromoxynil 500μM and linuron 500μM for 24h while "-" correspond to DMSO treated samples.

**
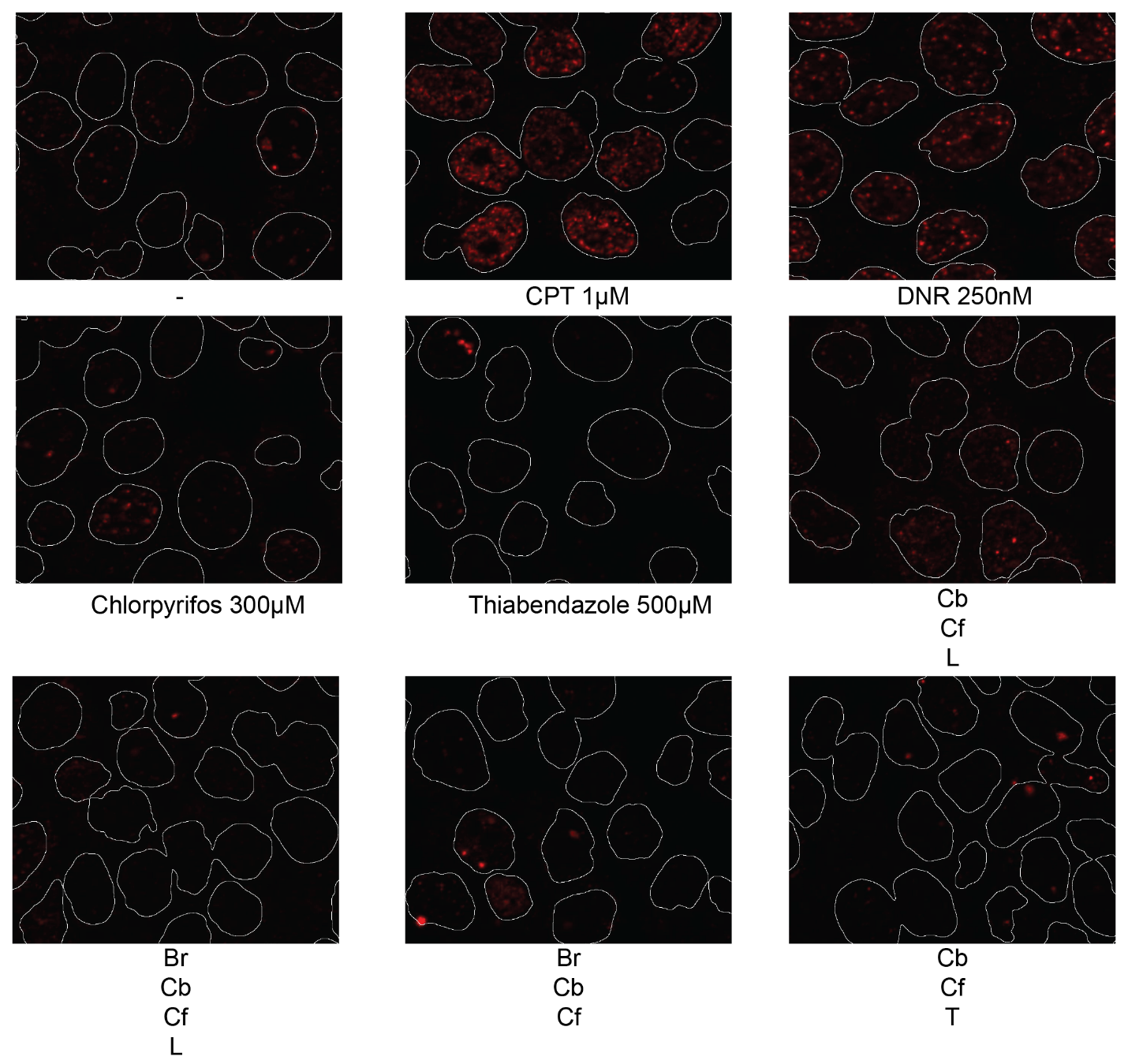
**

**Supplementary figure 5. Chlorpyrifos, thiabendazole and pesticide combinations at low concentrations do not cause DNA damage.**

γ-H2A.X foci were observed by immunofluorescence on MCF-7 cells grown in DMEM media supplemented with FBS 10 % for 48h and treated with DMSO, camptothecin 1μM (CPT), daunorubicin 250nM (DNR), chlorpyrifos 300μM, thiabendazole 500μM and bromoxynil 500μM and four combinations containing 20μM carbaryl, 40μM chlorpyrifos and 25μM linuron (Cf,Cb,L), or 60μM bromoxynil, 10μM carbaryl, 20μM chlorpyrifos and 12.5μM linuron (Cf,Cb,L,B), or 1μM carbaryl, 2μM chlorpyrifos and 60μM bromoxynil (Cf, Cb, B) and 1μM carbaryl, 2μM chlorpyrifos and 1μM thiabendazole (Cf, Cb,T) for 24h while "-" correspond to DMSO treated samples.
